# Editorial: Ubiquitin proteasome system (UPS) and ubiquitin-independent proteasome-mediated proteolysis (UIPP) crosstalk in development and disease

**DOI:** 10.3389/fcell.2025.1748172

**Published:** 2025-12-10

**Authors:** Michal Sharon, Ralf Stohwasser

**Affiliations:** 1 Department of Biomolecular Sciences, Weizmann Institute of Science, Rehovot, Israel; 2 Institute of Biotechnology, Biochemistry, Faculty of Environmental and Natural Sciences and Faculty of Health Science Brandenburg (coopted), Brandenburg University of Technology, Cottbus-Senftenberg, Senftenberg, Germany

**Keywords:** aging, caspase-like activity, proteasome, condensates, signalosome, neurodegeneration, stress granules, ubiquitin-independent proteasomal protein degradation

The Ubiquitin–Proteasome System (UPS) and Ubiquitin-Independent Proteasomal Protein Degradation (UIPP) represent the two principal proteasome-based pathways of intracellular protein turnover. These processes are intricately interconnected through their shared use of distinct 20S proteasome isoforms, including the constitutive (c20S), immunoproteasome (i20S), and tissue-specific variants, and their regulation by diverse proteasome activators (PAs) and inhibitors (PIs). Such diversity underlies functional specialization across cellular contexts, from non-lymphatic and interferon-untreated tissues (c20S) to lymphatic or interferon-γ-induced environments (i20S), and even to specialized forms such as neuron-associated membrane proteasomes or thymus-specific complexes (t20S). While multiple recent Frontiers Research Topics and other publications have extensively explored facets of the UPS, the UIPP and its crosstalk with the UPS, particularly under cellular stress and disease conditions, remain less well understood. The four contributions in this Research Topic aim to advance our understanding of these intertwined pathways and their roles in maintaining proteostasis.

In their opinion article, Dubiel and Dubiel present an insightful comparison between the LID complex of the proteasomal 19S regulatory particle and the COP9 signalosome (CSN). Both complexes share remarkable evolutionary and structural similarities, most notably between RPN11 in the LID and its paralog CSN5 in the CSN, each containing a metallo-deubiquitylase active site. While RPN11 removes ubiquitin chains from proteasome-bound substrates, CSN5 mediates deneddylation of cullins, thereby controlling the activity of cullin–RING E3 ubiquitin ligases (CRLs). These complexes exhibit a striking one-to-one correspondence across most subunits and share a comparable overall architecture that extends beyond their canonical deubiquitylating and deneddylating functions. Building on this analogy, the authors raise an intriguing question: could these signalosome–proteasome paralogs reciprocally regulate each other’s activity? Addressing this possibility will require a better understanding of the dissociation dynamics and subunit exchange between LID and CSN. Indeed, activity assays and co-immunoprecipitation studies have demonstrated partial interchangeability between the two complexes *in vitro*; however, whether the LID can substitute for the CSN under physiological conditions remains to be determined.

The involvement of UIPP degradation in neurodegenerative disorders is comprehensively reviewed by Church and Margolis. Their concise review focuses on UIPP mechanisms and the emerging roles these pathways play in the context of neurodegeneration. The authors discuss the diversity of ubiquitin-independent proteasomal pathways, emphasizing substrate recognition, targeting, the contribution of regulatory cofactors such as PA700, PA28, PA200, PI31, and the recently identified CCR family, which allosterically modulate uncapped 20S proteasome activity. Specialized proteasome assemblies including the immunoproteasome (i20S), neuronal membrane proteasomes, extracellular proteasomes, and hybrid proteasome complexes are also explored. These systems are examined within the broader context of aging, oxidative stress, protein aggregation, and age-associated neurodegenerative diseases, with particular attention to Alzheimer’s, Huntington’s, and Parkinson’s diseases. The review underscores that a mechanistic understanding of UIPP function and regulation in neuronal health and degeneration is essential for developing novel therapeutic strategies aimed at selectively targeting this degradation pathway in neurodegenerative disease.

Over the past decade, the study of protein condensates has attracted considerable attention. These non-membrane-bound, dynamic pseudo-compartments arise through the aggregation or phase separation of proteins within the cytoplasm or nucleus. Two articles in this Research Topic address protein turnover within such condensates, including liquid–liquid phase-separated structures like stress granules (SGs) and proteasome condensates. These so-called non-canonical UPS (nUPS) assemblies are associated with a wide range of stress conditions, such as nanoparticle exposure, inflammation, senescence, DNA damage, hyperosmotic stress, and amino acid starvation as reviewed by Ernst and Enenkel. Proteasome-containing condensates often include additional UPS components and may harbor condensate-specific proteolytic systems. They have been identified at the nuclear periphery and in the cytoplasm of mammalian cells, as well as in yeast, where they form proteasome storage granules (PSGs) that are reversible and contribute to stress resistance and enhanced cellular fitness during aging. Recent studies on proteasome condensates link their formation and dynamics to metabolic changes, suggesting an adaptive role of proteasome organization in maintaining proteostasis under stress.

Proteasome activities serve distinct and context-dependent roles in maintaining cellular homeostasis. Historically, these activities were characterized using fluorogenic peptide substrates, but more recent studies employing physiological protein substrates and mass spectrometry have revealed a more nuanced picture of their functions. Steinberger et al. explore the functional relevance of the caspase-like activity and its role in cellular adaptation to stress. Using engineered cell lines lacking this specific activity, the authors show that the mutant cells display slower proliferation, heightened stress sensitivity, and activation of the unfolded protein response (UPR), as evidenced by elevated stress markers. This observation is particularly intriguing, as the chymotrypsin-like activity is typically considered the dominant and rate-limiting. Moreover, associated regulatory particles are known to allosterically influence the catalytic properties and cleavage preferences of the 20S core, further shaping proteasomal activity. The findings by Steinberger and colleagues thus add a valuable cell-biological dimension to our understanding of proteasome regulation, emphasizing the specialized contributions of individual proteolytic activities to cellular stress responses and protein quality control.

In summary, the four contributions presented in this Research Topic highlight the growing complexity and versatility of proteasome biology. Collectively, they emphasize the multifaceted regulation of proteasomal activity and its central role in sustaining cellular proteostasis across diverse physiological and pathological contexts. This Research Topic reinforces the view that the proteasome is far from a static proteolytic machine; rather, it is a highly dynamic and adaptable system, continually reshaped by cellular context, stress, and evolution, remaining an enduring and fertile area of discovery in cell biology.

